# Clinical characteristics and prognosis of anti-γ-aminobutyric acid-B receptor encephalitis: A single-center, longitudinal study in China

**DOI:** 10.3389/fneur.2022.949843

**Published:** 2022-09-15

**Authors:** Xuedan Feng, Yujing Zhang, Yu Gao, Jing Zhang, Shasha Yu, Jing Lv, Yu Zu, Lin Wang, Xiangbo Wang

**Affiliations:** ^1^Department of Neurology, Beijing Fengtai You'anmen Hospital, Beijing, China; ^2^Department of Neurology, Xuanwu Hospital of Capital Medical University, Beijing, China

**Keywords:** anti-γ-aminobutyric acid-B receptor encephalitis, autoantibody, lung cancer, seizure, prognosis

## Abstract

**Objective:**

Anti-γ-aminobutyric acid-B receptor (GABA_B_R) encephalitis is a rare type of autoimmune encephalitis. There are only a few, small, published studies regarding prognosis, so prediction of prognosis is of limited accuracy. We identified 37 cases of anti-GABA_B_R encephalitis in China. Here, we present these patients' clinical characteristics and long-term outcomes.

**Methods:**

We collected and retrospectively analyzed the clinical data of 37 patients with anti-GABA_B_R encephalitis from Beijing Fengtai You'anmen Hospital.

**Results:**

The study cohort comprised 37 patients of anti-GABA_B_R encephalitis of median age 61 years (range: 11–77), 28 of whom were male. The main clinical manifestations were epilepsy (91.9%, 34/37), psychiatric disorders (94.6%, 35/37) and cognitive impairment (97.3%, 36/37). Tumors were identified in 18 (48.6%) patients. First-line immunotherapy was administered to 34 patients, 31 of whom (90.6%) responded favorably. During a median follow-up of 18 months (range: 1–72 months), 21 patients had good outcomes [Modified Ranking Scale (mRS ≤2)], 16 (43.2%) died (mRS 6), and 7 (18.9%) relapsed. Age (*P* = 0.005), disturbance of consciousness (*P* = 0.018), admission to the Neurology Intensive Care Unit (*P* = 0.003), mechanical ventilation (*P* = 0.009), more numerous clinical manifestations (*P* = 0.008), comorbid malignancy (*P* = 0.008), multiple anti-neuronal antibodies (*P* = 0.029), and hyponatremia (*P* = 0.023) differed significantly between patients with good outcomes (mRS 0–2) and those with poor outcomes (mRS 3–6).

**Conclusion:**

Men aged 50–70 years accounted for most of the patients with anti-GABA_B_R encephalitis in our case series. The main clinical manifestations were epilepsy and neuropsychiatric dysfunction. The participants often had concomitant lung cancer, particularly small-cell lung cancer. Patients with lung tumors and/or serious manifestations usually had a poor prognosis with high mortality. Early identification and treatment of tumors improved the poor prognosis to some extent.

## Introduction

Anti-γ-aminobutyric acid-B receptor (GABA_B_R) encephalitis is caused by specific autoimmune antibodies against GABA_B_R on the surfaces of neurons, preventing γ-aminobutyric acid (GABA) from exerting neuroinhibitory effects (1). The main clinical manifestation is evidence of limbic encephalitis such as seizures, memory loss, mental behavioral abnormalities, and disturbance of consciousness (1, 2). Since anti-GABA_B_R encephalitis was first reported in 2010 by Lancaster et al. (2), various cases have been reported in eastern and western countries. Because this type of encephalitis is not commonly encountered in clinical practice (2, 3), few studies have been published on it (1–16). The current study investigated the clinical characteristics, autoantibody profiles, responses to treatment, and prognosis in 37 patients with anti-GABA_B_R encephalitis.

## Methods

### Patients

The cohort of this retrospective study comprised consecutive patients diagnosed with anti-GABA_B_R encephalitis [including 21 patients previously reported (17)] at the Department of Neurology of Beijing Fengtai You'anmen Hospital between October 2012 and July 2022. Inclusion criteria were as follows: (i) diagnoses made in accordance with the criteria for autoimmune encephalitis published by Graus et al. in 2016 (18), namely positive anti-GABAB receptor antibody, at least one neurological or psychiatric symptom, at least one abnormal finding on auxiliary examination (examination of cerebrospinal fluid [CSF], neuroimaging or electrophysiological examination), or associated tumors; and (ii) follow-up for more than 6 months. Exclusion criteria were as follows: (i) anti-intracellular antigen-antibody encephalitis and other types of encephalitis of unknown origin; and (ii) follow-up for <6 months or lost to follow-up. This study was approved by the Local Ethics Committee.

### Clinical information

The clinical data collected included patient characteristics, clinical manifestations, results of laboratory tests (serum and CSF), findings on electroencephalogram (EEG), brain computed tomography (CT)/magnetic resonance imaging (MRI) and chest CT, treatment protocols, response to treatment, and follow-up data. Anti-neuronal antibodies detected in serum and/or CSF samples by indirect immunofluorescence assay (Euroimmun AG, Luebeck, Germany) included anti-GABAB receptor (neuronal surface antigen) and anti-Yo, -Hu, -Ri, -CV2/CRMP5, -amphiphysin, -Ma2/Ta and -GAD 65 antibodies (intracellular antigens).

### Treatment and prognosis

The treatment protocols were mainly immunotherapy and supportive treatment such as anti-epilepsy, anti-infection, and tranquilizing medications. First-line immunotherapy included glucocorticoids and intravenous immunoglobulin (IVIg). Second-line immunotherapy, including mycophenolate mofetil, cyclophosphamide, or rituximab, was often used in patients who failed first-line immunotherapy or recurrence. The patients with tumors received chemotherapy alone or in combination with radiotherapy and/or surgery. Surgery could be performed on those patients with Limited-stage SCLC patients (T1-2, N0). All patients were followed up every 3 months at clinic visits or by telephone interviews. The following factors were evaluated during follow-up: (i) changes in symptoms; (ii) relapse, death, and cause of death; and (iii) modified Rankin Scale (mRS) scores (19). These factors were evaluated on admission, one week after immunotherapy, and every 3 months after the end of immunotherapy. The follow-up period was defined as the interval from discharge to the patient's death or last follow-up. Patients were divided into two groups (favorable vs. poor outcomes) on the basis of their mRS scores at the last follow-up. Good outcomes were defined as mRS scores of 0–2 and poor outcomes as mRS scores 3–6. Relapse was defined as recurrence of symptoms after disease improvement or stabilization for more than 2 months or exacerbation of symptoms (mRS score increased by more than one point).

### Statistical analysis

Statistical analysis was performed using SPSS version 23.0 (IBM Corp., Armonk, NY, USA). Descriptive statistics were used to analyze clinical data; these are expressed as median or mean values and percentages. Continuous variables that were normally distributed are expressed as mean ± standard deviation and were compared using Student's *t*-test.

Continuous variables that were not normally distributed are expressed as the median (m) and were compared using the Mann–Whitney *U*-test. Fisher's exact test was used to compare categorical variables. Differences were considered statistically significant when *P* < 0.05.

## Results

### Clinical characteristics

We identified 37 patients who had been diagnosed with anti-GABA_B_R encephalitis. Their median age was 61 years (range: 11–77) and 28 were male. Nine patients (9/37) had prodromal symptoms within the 2 weeks before onset (three had headaches, two colds, one otitis media, two fever, and one fatigue). Additionally, the initial manifestation was numbness and weakness in the left upper limb in one patient, mental and behavioral disorder in two, fever in one, speech impairment in two, and intellectual impairment in three. The initial manifestation in the remaining 28 patients was seizures.

**Figure 1:**
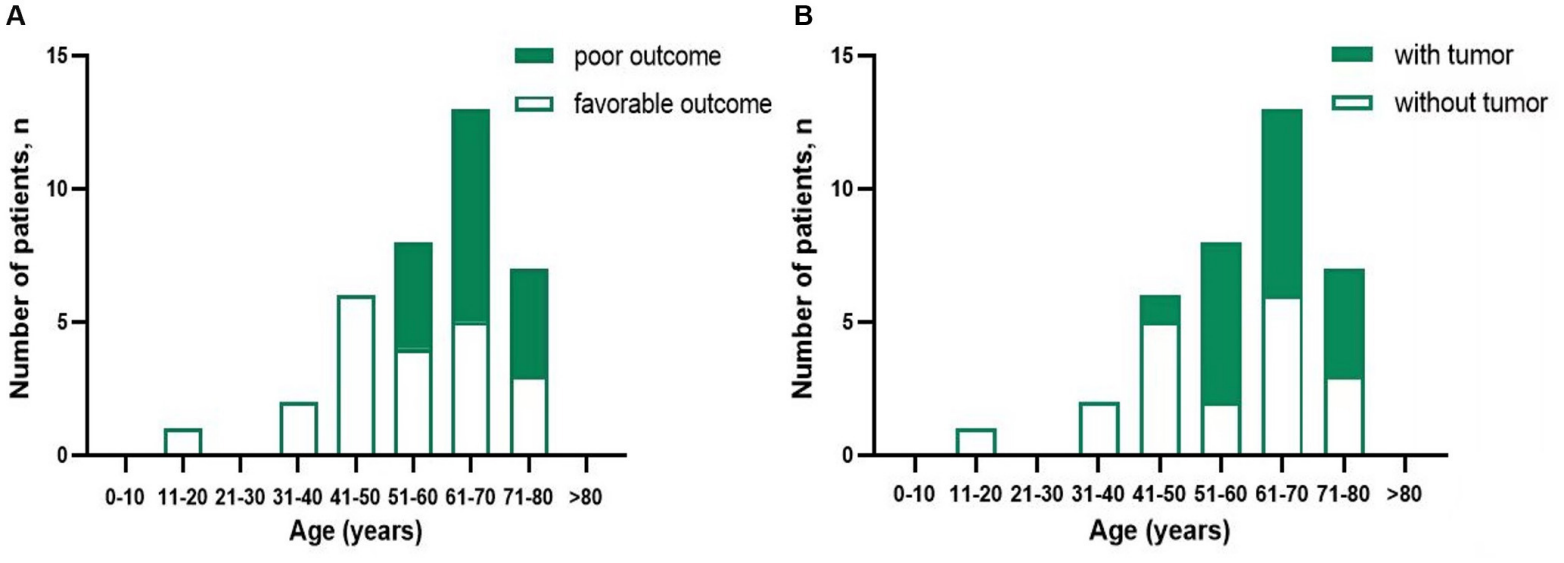
Distribution of prognosis patients with anti-GABA_B_R encephalitis **(A)** and presence of tumor **(B)** according to age.

Clinical manifestations included epilepsy, cognitive impairment, psycho-behavioral disturbances, language disorder/aphasia, sleep disorders, disturbance of consciousness, involuntary movements, autonomic nerve dysfunction, and ataxia. The patients' relevant clinical characteristics are summarized in Table 1 and Figure 1. The median interval between first manifestation and diagnosis was 22 days (range: 5–435 days). Lung cancer was diagnosed by pathological examination of biopsy specimens in 18 cases (48.6%); these cancers comprised 17 small-cell lung cancers (SCLC) and one large-cell neuroendocrine carcinoma of the lung. The median interval between first clinical manifestation and diagnosis of a tumor was 4 months (range: −3 to 20 months).

**Table 1:** Clinical data of patients with anti-GABA_B_R encephalitis according to outcome.

**Variable**	**Total (*n* = 37)**	**Favorable outcome (*n* = 21)**	**Poor outcome (*n* = 16)**	***P-*value**
**Age (years)**	61 (11–77)	57 (11–73)	63.5 (55–77)	0.005
**Sex (** ***n*** **, %)**				
Male	28	15 (66.7)	13 (87.5)	0.702
Female	9	6 (28.6)	3 (18.8)	
**Symptoms (** ***n*** **, %)**				
Seizures	34	19 (90.5)	15 (93.8)	1.000
Psychiatric disorder	35	19 (90.5)	16 (100.0)	0.495
Cognitive impairment	36	20 (95.2)	16 (100.0)	1.000
Speech impairment	27	16 (76.2)	11 (68.8)	0.716
Consciousness disturbance	21	8 (38.1)	13 (81.3)	0.018
Ataxia	1	0 (0.0)	1 (6.3)	0.432
Sleep disorders	28	15 (71.3)	13 (81.3)	0.702
Involuntary movement	12	5 (23.8)	7 (43.8)	0.291
Autonomic dysfunction	10	3 (14.3)	7 (43.8)	0.067
**Time from symptom onset to diagnosis (month)**	23 (5–435)	32 (5–435)	21 (7–150)	0.291
**Admission to ICU**	17	5 (23.8)	12 (75.0)	0.003
**Mechanical ventilation**	16	5 (23.8)	11 (68.8)	0.009
**Complication with tumors**	18	6 (28.6)	12 (75.0)	0.008
**Disease severity**				
mRS score at the peak of disease	4 (2–5)	3 (2–5)	4 (2–5)	0.107
Number of symptoms	5 (3–9)	5 (3–8)	6.5 (4–9)	0.008
Pulmonary infection	29	14 (66.7)	15 (93.8)	0.104
Relapse	7	3 (14.3)	4 (25.0)	0.437
**CSF analysis**				
Increased level of WBC	13	10 (47.6)	3 (18.8)	0.091
Increased level of protein	9	5 (23.8)	4 (25.0)	1.000
Pressure >180 mmH_2_O	5	3 (14.3)	2 (12.5)	1.000
**Serum analysis**				
Hyponatremia	16	5 (23.8)	10 (62.5)	0.023
Increased level of tumor markers^a^	27	15 (71.4)	12 (75.0)	1.000
**Additional antibodies**	7	1 (4.8)	6 (37.5)	0.029
**MRI abnormality in limbic system**	20	11 (52.4)	9 (56.3)	1.000
**EEG abnormality^b^**	25	16 (88.9)	9 (75.0)	0.364
**Abnormal pulmonary CT**	33	17 (78.9)	16 (100.0)	0.118
**No immunotherapy**	3	0 (0.0)	3 (18.8)	0.072
**First-line immunotherapy**	34	21 (100.0)	13 (81.3)	0.072
Steroids	3	2	1	
IVIg	5	4	1	
Steroids + IVIg	26	15	11	
**Second-line immunotherapy**	5	4 (19.0)	1 (6.25)	0.364
**Time to initiating immunotherapy after symptom onset^c^**				
≤ 4weeks	21	12 (57.1)	9 (56.3)	0.718
>4weeks	13	9 (42.9)	4 (25.0)	
**Tumor therapy**	12	6 (100.0)	6 (50.0)	0.054

Quantitative variables are expressed as the median and range and categorical variables as numbers and percentages of participants. Favorable outcomes were defined as mRS score 0–2 and poor outcomes as mRS scores 3–6 on last follow-up.

ICU, intensive care unit; mRS, modified Rankin Scale; WBC, white blood cell; IVIg, intravenous immunoglobulin.

a Analysis of 31 patients with results available.

b Analysis of 30 patients with results available.

c Analysis of 34 patients with results available.

### Auxiliary investigations

Thirty-seven patients were positive for anti-GABA_B_R antibodies in CSF and serum. Seven patients (18.9%, 7/37) were positive for additional antibodies, including anti-Hu, SOX1, collapsin response mediator protein 5 (CV2), and anti-glutamic acid decarboxylase 65-kD isoform antibody. Anti-herpes simplex virus type 1 (HSV-1) IgG antibody (+) was detected in the CSF in two patients.

Of the 31 patients who underwent EEG examination, 26 showed abnormalities (83.9%), 19 of which were moderate to severe (61.3%). Most of these patients had focal or diffuse slow waves, mostly in the frontotemporal lobe. Additionally, abnormal epileptic discharges were identified in six patients (19.4%).

All patients underwent a cranial MRI. In 26 patients (70.3%), this revealed high signals on T2-weighted imaging (T2WI) and fluid-attenuated inversion recovery sequences that were mainly located in the medial temporal lobe (including hippocampus; 54.1%), followed by the frontoparietal lobe, paraventricular region, occipital lobe, corpus callosum, and semioval center. Pulmonary CT of 33 of the patients (89.2%) was abnormal, findings including pulmonary infection, pulmonary space-occupying lesions, hilar mediastinal lymph node enlargement, pleural effusion, atelectasis, and pulmonary embolism. The results of these auxiliary investigations are shown in Table 1.

### Treatment and follow-up

After hospitalization, first-line immunotherapy was administered during the acute phase to 34 patients (91.9%), 26 patients (70%) treated with a combination of IVIg and steroids, 8 patients (21.6%) received steroids or IVIg therapy alone, 5 patients (13.5%) received second-line immunotherapy and 3 patients (8.1%) were not given immunotherapy. The median interval between symptoms onset and immunotherapy was 24.5 days (range: 8 days–16 months). Eleven of the 18 patients with lung cancer received chemotherapy alone or in combination with radiotherapy; one patient had attempted surgical resection and postoperative radiotherapy.

**Figure 2:**
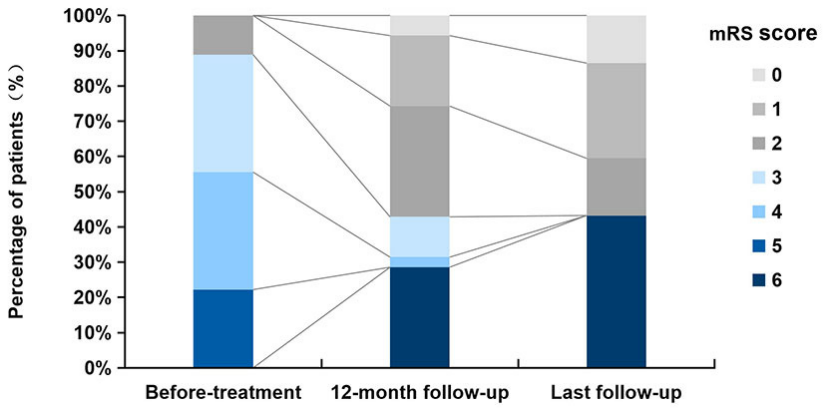
Distribution of mRS scores before treatment, at 12-month follow-up, and at last follow-up.

During the first 12 months, twenty patients (20/35, 57.1%) had neurological improvement and reached an mRS 0–2 points; five patients (5/35, 14.3%) reached an mRS 3–5 points; ten patients (10/35, 28.6%) deteriorated and eventually died (mRS 6 points). At the last follow-up, twenty-one patients (56.8%) had favorable outcomes (mRS ≤ 2), including six with lung cancer whose median follow-up was 36.5 months (range: 6–72), the main manifestations being a mild cognitive impairment, neuropsychiatric abnormalities, and epilepsy. Sixteen patients had a poor prognosis (mRS 6), including twelve with neoplastic anti-GABA_B_R encephalitis (32.4%), whose median interval between initial manifestations and death was 14.5 months (range: 3–24 months). Neoplastic complications were the leading cause of death; the remaining four patients (10.8%) presented with severe neurological symptoms and multiple organ damage or failure; although they did not diagnose with tumors, two died of pulmonary infection, one died of multiple organ dysfunction syndromes, one was unclear. The median interval between initial manifestations and death was 5 months (2–12 months). The mRS scores in our series before immunotherapy, at the 12-month follow-up, and the last follow-up are shown in Figure 2.

Recurrence occurred in seven patients (18.9%), all of whom developed frequent and uncontrollable epilepsy, two of them dying of multiple organ dysfunction syndromes secondary to status epilepticus. The median time to recurrence was 6 months (range: 2–32).

### Analysis of prognostic factors

Possible prognostic factors in patients with anti-GABA_B_R encephalitis were analyzed. Characteristics of patients with favorable vs. poor outcomes are summarized in Table 1. We found that the poor outcome group was significantly older than the good outcome group (*P* = 0.005). There were also significant differences between these two groups in rates of disturbance of consciousness (*P* = 0.018), admission to the intensive care unit (ICU; *P* = 0.003), mechanical ventilation (*P* = 0.009), presence of a tumor (*P* = 0.008), and the number of clinical manifestations (*P* = 0.008). Additionally, multiple anti-neuronal antibodies (*P* = 0.029) and hyponatremia (*P* = 0.023) were associated with a poor prognosis in our study cohort.

**Figure 3:**
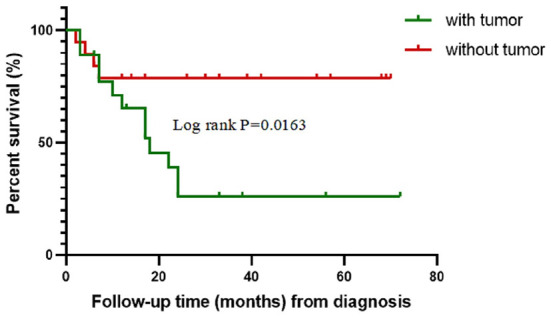
Kaplan–Meier survival curves for 37 patients with anti-GABA_B_R encephalitis.

We further conducted a survival analysis of patients with (48.6%, 18/37) and without tumor (51.4%, 19/37) to assess the impact of tumors on prognosis (Figure 3). Overall survival (OS) was defined as the interval between diagnosis of anti-GABA_B_R encephalitis and death or last follow-up. The OS rate of patients with tumors was significantly lower than that of patients without them (log-rank *P* = 0.0163). The estimated median survival time of patients with tumors was 14.5 months and without tumors could not be determined.

## Discussion

The study cohort comprised 37 patients with anti-GABA_B_R encephalitis. Some important characteristics of the current series are as follows: (i) To the best of our knowledge, this is the largest reported, single-center, retrospective, cohort study of anti-GABA_B_R encephalitis and has the greatest age span and longest follow-up of any study in China (3–6, 8, 11–16). (ii) In this group, two patients had anti-GABA_B_R encephalitis secondary to HSV-1 infection. (iii) Multiple anti-neuronal antibodies were detected in seven patients in our series. (iv) We also found that hyponatremia as a prognostic factor in anti-GABA_B_R encephalitis.

A systematic review revealed an average onset age of anti-GABA_B_R encephalitis of 55.2 years (range: 18–76) in Asian patients and that 62.3% of them are male (16). The average age of onset in the present series was 61 years (range: 11–77) and the male: female ratio 28:9, which is consistent with the ages of onset that have been reported in western countries. However, the percentage of male patients is reportedly higher in those countries (1, 2, 7, 9, 10, 20). As previously reported (2–5, 9), in our series older age (>50 years) was associated with a poor prognosis, this being related to the high incidence of malignant tumors in older persons. The rate of concomitant malignant tumors was 60.7% (17/28) in patients aged over 50 years, whereas it was only 11.1% (1/9) in patients younger than 50 years.

There is characteristically no clear history of infection before the onset of anti-GABA_B_R encephalitis (21). However, there may be prodromal symptoms such as headache and influenza-like symptoms; nine of our study patients had headaches and fever before onset. Additionally, there are some novel findings in our cohort: (i) One patient in this cohort was an 11-year-old boy with congenital “language retardation”; this may have denoted a genetic susceptibility to anti-GABA_B_R encephalitis. Further investigation of this possibility is needed. (ii) Two patients in the present series had sustained trauma within the month before the onset of anti-GABA_B_R encephalitis. Whether trauma is linked with this disease is unknown. (iii) two cases had anti-GABA_B_R encephalitis secondary to HSV-1 encephalitis, indicating that HSV-1 infection of the central nervous system can trigger anti-GABA_B_R encephalitis (22). Sometimes multiple factors contributed to the development of anti-GABA_B_R encephalitis. For example, One case had anti-cytomegalovirus antibody (IgG) and HSV-1 antibody IgG positivity in the CSF and meningeal irritability, and lung cancer was diagnosed 4 months later.

Anti-GABA_B_R encephalitis usually starts with epilepsy. Consistent with this, the most prominent features in the early stage are various types of epileptic seizures and neuropsychiatric symptoms (2–7, 9, 10, 12). The latter mostly manifest as personality changes (anxiety, depression, mania), loss of memory for recent events, behavioral abnormalities, disorders of consciousness, and sleep disorders (1–3, 5–7, 9, 10). When the brain stem, cerebellum, and other regions are involved, patients may present with opsoclonus myoclonus syndrome, ataxia, chorea, and brainstem encephalitis (1, 6, 21, 23). In our series, the clinical manifestations were similar to those reported previously. One patient presented with cerebellar ataxia, which was consistent with the distribution of GABA_B_R in the cerebellum (23)_._ We found that the mental and behavioral abnormalities were milder and less long-lasting in this series than has been reported for patients with anti-NMDAR encephalitis. Respiratory complications are common in patients anti-GABA_B_R encephalitis, more than 2/3 of patients having pulmonary infections (4). Pulmonary infection and respiratory failure are reportedly predictors of poor prognosis (3, 5, 24). The incidence of pulmonary infection in the present study was 78.4%; however, we did not find a significant relationship with prognosis. In the present series, impairment of consciousness occurred in 21 patients (56.8%). As a consequence of status epilepticus, pulmonary infection, and sedative drugs, seventeen patients (45.9%) required admission to ICU. The ICU occupancy rate in our group was higher than the 22.2% (4/18) (6) and 10.7% (3/28) (4) previously reported in China, our rate of ICU admission being closer to the 64% reported abroad (10). Disturbance of consciousness, admission to ICU and mechanical ventilation may be important indicators of poor prognosis. Furthermore, in our series patients with poor outcomes had more numerous clinical manifestations than those with good outcomes.

More than half of patients with anti-GABA_B_R encephalitis have concurrent tumors, most of which are SCLC (2–7, 9, 12–15), other associated tumors include thymoma (25), esophageal cancer (1), and melanoma (23), and so on. While only 40/114 (36%) of patients in an Asian series had tumors (13, 16). Eighteen of our patients (48.6%) were diagnosed with lung cancer, comprising one large-cell neuroendocrine carcinoma of the lung and 17 SCLC. The low prevalence of tumors in some Chinese studies may be attributable to more youthful enrolled cases, small series, insufficient screening for tumors, and relatively short follow-up times (12).

Consistent with previous studies (4, 6, 7), we found strong evidence for tumors being critical prognostic factors in patients with anti-GABA_B_R encephalitis. Such tumors are usually identified within 6 months of the onset of that encephalitis (1, 2). One patient in our cohort was diagnosed as having lung cancer 3 months before the onset of encephalitis-related symptoms, the remaining tumors being diagnosed after the onset: 14 within 6 months, two after 12 months, and one after 20 months. Therefore, all patients with anti-GABA_B_R encephalitis should be followed up and encouraged to undergo regular screening for tumors, especially lung cancer. It is recommended that such screening be performed at 3-monthly intervals for at least 2 years. SCLC is often associated with paraneoplastic neurological syndromes, one of which is inappropriate secretion of anti-diuretic hormone, leading to intractable hyponatremia (26). Anti-GABA_B_R encephalitis patients with tumor were more likely to develop hyponatremia (13). In the present study, significantly more patients with (50.0%, 9/18) than without (21.1%, 4/19) tumors developed hyponatremia. Our finding suggests hyponatremia maybe one presentation of paraneoplastic syndrome and was also associated with poor prognosis. However, because ours was a small study, further studies are needed to confirm this finding.

Various anti-neuronal antibodies in addition to anti-GABA_B_R are reportedly found in 7%−40% of patients with anti-GABA_B_R encephalitis (5, 6). It has been found that the anti-Hu antibody is the commonest additional antibody, this antibody being strongly associated with SCLC (9, 21, 23). Anti-SOX1 (27) and anti-CV2 (28) antibodies are also associated with SCLC. In our study cohort, 7/37 (18.9%) patients had one or more above antibodies. Six patients who were positive for additional anti-neuronal antibodies had concomitant lung cancer. The malignant tumor was not detected in the last patient during six-month follow-up. The presence of additional anti-neuronal antibodies predicts a poor prognosis in patients with anti-GABA_B_R encephalitis. One patient in our cohort had three different anti-neuronal antibodies, namely anti-GABA_B_R, anti-GAD65, and anti-CV2 antibodies; this combination of antibodies is rare. The prevalence of anti-CV2 antibody in patients with autoimmune encephalitis is reportedly only 0.7/100,000 (28). This patient was characterized by SCLC, intractable epilepsy, refractory hyponatremia and cognitive impairment.

Studies have shown that neurological deficits improve in about 90% of patients with anti-GABA_B_R encephalitis receiving immunotherapy: indeed, in 50% of them neuropsychiatric manifestations resolve completely (25). Most patients in this group who received first-line immunotherapy responded well to the treatment, regardless of whether or not they had a tumor. The choice of immunotherapeutic agents mainly considers the severity of the disease, the patient's economic situation, and the tolerance of steroids. In this group, three patients gave up immunotherapy due to economic constraints; the first one was critical and deteriorated rapidly to death; the second one died of unclear cause within 14 months with the likelihood of an underlying tumor; the neurological status of the last one improved after receiving chemotherapy, while eventually died of neoplastic complications within 23 months. Our results were consistent with the previous findings that there was no significant correlation between the time of initial immunotherapy and the prognosis (5, 13). It was not rare to be misdiagnosed as epilepsy, viral encephalitis, and psychosis for patients, partly due to autoimmune encephalitis (AE)-related antibodies detection having not been widely popularized; in addition, some patients were easy to be ignored as their symptoms were mild or atypical. In the current study, six patients with malignant tumors had favorable prognoses, all of whom were diagnosed with limited-stage SCLC and responded well to the tumor treatment. Early diagnosis and active treatment of tumors may be vital to improving the prognosis of anti-GABA_B_R encephalitis (10, 13). About 10.1%–21.0% of patients relapsed (4, 5, 24). Seven patients in our group had a relapse, five with malignant tumors, which indicated that concurrent malignancies might be a risk factor for disease recurrence. The possibility of tumor recurrence or potential tumor during the initial episode should be considered in patients with a relapse. However, we did not identify significant relationships between recurrence and tumor or prognosis.

The prognosis of patients with anti-GABA_B_R encephalitis varies greatly, mainly depending on whether there is an associated tumor (4, 6). In this study, most patients without tumors (15/19) had good outcomes with no obvious or mild sequelae, whereas most of the patients with tumors (12/18) had serious manifestations, a poor prognosis, and high mortality. The mortality of our cohort (51.4%) was slightly higher than the 9.1%−32.1% previously reported in China (4, 6, 8, 11, 12), and closer to that reported abroad (2, 7, 10). This discrepancy may at least in part be attributable to the longer duration of follow-up in our study. During the 1-year follow-up, the patients with non-neoplastic anti-GABA_B_R encephalitis reached a relatively stable stage, while some neoplastic patients still died in the second year. Consistent with previous reports (4), the most prolonged interval from initial symptoms to death was 24 months indicating the disease seems to reach a plateau two years later. We found that patients older than 50 years with intractable hyponatremia and additional anti-neuronal antibodies constituted a high-risk subgroup of patients of anti-GABA_B_R encephalitis with underlying malignant tumors. We also found that patients with serious manifestations of anti-GABA_B_R encephalitis usually had a poor prognosis. These patients usually had multiple clinical manifestations, concurrent malignancies, disturbance of consciousness, admission to NICU, and mechanical ventilation. Additionally, some patients with anti-GABA_B_R encephalitis secondary to HSV-1 encephalitis can occur severe and persistent brain damage and even multiple organ dysfunction syndrome, which can lead to poor prognosis. So far, to our knowledge no researchers have reported a link between HSV-1 infection and a poor prognosis of anti-GABA_B_R encephalitis.

Our study had several limitations. Firstly, it was a relatively small, single-center, retrospective study. Secondly, some patients had a short follow-up period or incomplete data. Thirdly, we could not perform multiple regression analysis because of the small sample size. Therefore, large-scale, prospective, multicenter research is needed to further explore our findings.

In conclusion, in our cases series anti-GABA_B_R encephalitis occurred more frequently in men aged 50–70 years. The main clinical manifestations were epilepsy and neuropsychiatric dysfunction. About half the patients had concomitant lung cancer, particularly SCLC. Most patients responded well to first-line immunotherapy. Most patients with anti-GABA_B_R encephalitis and a poor prognosis had an associated complicating malignancy and/or serious manifestations or their disease was secondary to HSV encephalitis. Early identification and treatment of tumors can improve the poor prognosis to some extent. It is recommended that patients with anti-GABA_B_R encephalitis be screened at 2–3 month intervals for at least 2 years.

## Data availability statement

The raw data supporting the conclusions of this article will be made available by the authors, without undue reservation.

## Ethics statement

The study was conducted with the approval of the Ethics Committee of Beijing Fengtai You'anmen Hospital (bjftyamyyll2021-8). Written informed consent from the participants' legal guardian/next of kin was not required to participate in this study in accordance with the national legislation and the institutional requirements. Written informed consent was obtained from the individual(s), and minor(s)' legal guardian/next of kin, for the publication of any potentially identifiable images or data included in this article.

## Author contributions

XF designed and conducted of the study. YZ and XF drafted and revised the manuscript. YG, JZ, and SY collected and analyzed the clinical data. JL and YZ performed the statistical analyses. LW and XW revised the manuscript. All authors contributed to the article and approved the submitted version.

## Funding

This study was supported by the National Natural Science Foundation of China (NSFC) (Grant No. 81901225).

## Conflict of interest

The authors declare that the research was conducted in the absence of any commercial or financial relationships that could be construed as a potential conflict of interest.

## Publisher's note

All claims expressed in this article are solely those of the authors and do not necessarily represent those of their affiliated organizations, or those of the publisher, the editors and the reviewers. Any product that may be evaluated in this article, or claim that may be made by its manufacturer, is not guaranteed or endorsed by the publisher.
